# Internal auditory meatus vascular loops and vestibulocochlear neurovascular contact on MRI: Are they associated with pulsatile tinnitus?

**DOI:** 10.1007/s00330-025-11660-8

**Published:** 2025-05-12

**Authors:** Mervyn L. Chong, Kyle R. S. Stephenson, Mehrshad Sultani Tehrani, Irumee Pai, Steve E. J. Connor

**Affiliations:** 1 Department of Radiology, St Bartholomew’s Hospital and Royal London Hospital, Barts Health NHS Trust, London, UK; 2 Department of Radiology, Guy’s Hospital and St Thomas’ Hospital, London, UK; 3 Faculty of Life Sciences and Medicine, King’s College London, London, UK; 4 School of Biomedical Engineering and Imaging Sciences, King’s College London, London, UK; 5 Department of Ear, Nose and Throat Surgery, Guy’s Hospital and St Thomas’ Hospital, London, UK; 6 Department of Neuroradiology, King’s College Hospital, London, UK

**Keywords:** Pulsatile tinnitus, Internal auditory meatus, Vascular loops, Neurovascular contact, Magnetic resonance imaging

## Abstract

**Objectives:**

To compare the presence of internal auditory meatus vascular loops (IVLs) or vestibulocochlear neurovascular contact (CN8-NVC) between unexplained unilateral pulsatile tinnitus (PT) ears and contralateral asymptomatic ears. Furthermore, to investigate whether IVL depth or angulation, or CN8-NVC location is associated with the presence of PT.

**Materials and methods:**

Single-centre retrospective case-controlled study of patients undergoing three-dimensional T2-weighted MRI for unexplained unilateral PT from January 2012 to July 2021. Two blinded observers recorded the presence of IVLs or CN8-NVCs, whilst evaluating IVL depth and angulation. Proportions of ears with IVLs or CN8-NVCs were compared between PT ears and contralateral control ears with McNemar’s test. Mann–Whitney *U* or Student’s *t*-test compared the depth and angulation of IVLs and the location of CN8-NVC with respect to the transition zone between ears with and without PT.

**Results:**

Three hundred thirty-seven patients were evaluated (250 female; mean age 47 ± 16 years). There was no significant difference between the proportion of IVLs (19.3% vs 25.2%; *p* = 0.06) or CN8-NVCs (59.9% vs 65.6%; *p* = 0.12) in PT ears as compared to contralateral control ears. There was no significant difference in IVL depth (median loop-fundus distance 6.5 mm vs 6.8 mm; *p* = 0.45), IVL angulation (median interlimb distance 3.1 mm vs 3.3 mm; *p* = 0.54), or CN8-NVC location within the transition zone (*p* = 0.58) between ears with and without PT.

**Conclusion:**

Unexplained unilateral PT is not associated with the presence of an ipsilateral IVL or CN8-NVC. Likelihood of PT is not influenced by depth or angulation of an ipsilateral IVL, nor by whether CN8-NVC is at the transition zone.

**Key Points:**

***Question*** *The relevance of internal auditory meatus (IAM) vascular loops or vestibulocochlear NVC in the context of PT remains uncertain*.

***Findings*** *Likelihood of unilateral PT was not influenced by the presence, depth, or angulation of IAM vascular loops, nor by the presence or location of vestibulocochlear NVC*.

***Clinical relevance*** *This study argues against the analysis and reporting of IAM vascular loops or vestibulocochlear NVC in the context of PT, and it gives the clinician confidence to reassure the patient that these are unlikely to be an aetiology*.

**Graphical Abstract:**

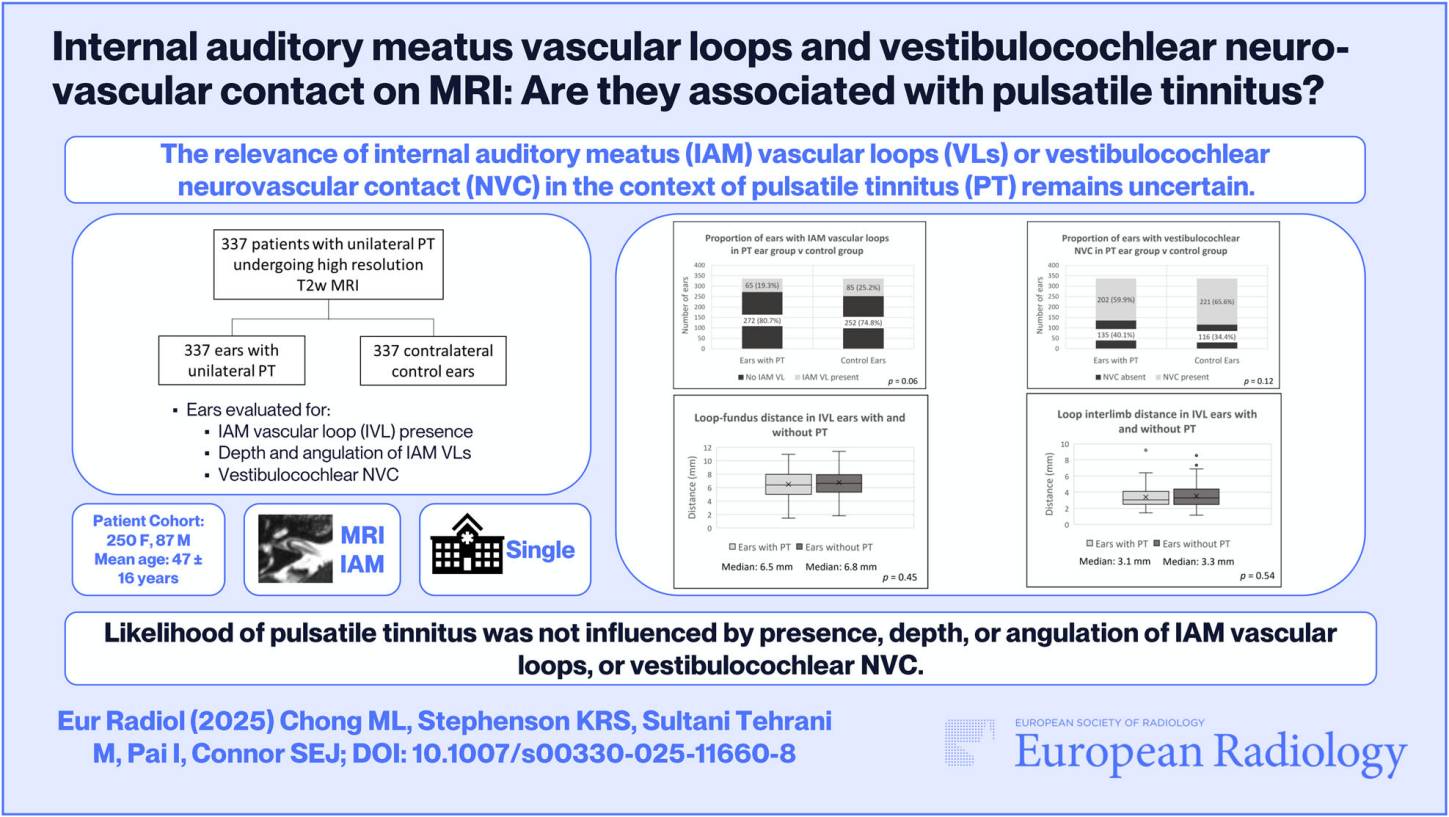

## Introduction

Tinnitus is the perception of sound in the absence of a corresponding external stimulus [1]. Pulsatile tinnitus (PT) is perceived as a rhythmic sound at the rate of the cardiac cycle and is thought to represent 4–10% of tinnitus [2, 3]. Imaging is performed in order to identify the aetiology of PT, which may aid prognostication and target therapy. Evaluating the diagnostic yield of imaging in PT is challenging due to the heterogeneity of study designs, with rates of positive findings ranging from 44% to 91% [4]. The most frequent abnormalities include paragangliomas, dural arteriovenous fistulae, idiopathic intracranial hypertension, venous anatomical variants and atheromatous disease [5–7]. Both CT and MRI-based protocols with vascular imaging are recommended for the investigation of PT [8].

Previous authors have proposed that normal internal auditory meatus (IAM) vascular loops (VLs) may also be responsible for PT [9, 10]. Proposed mechanisms include neurovascular contact (NVC) and demyelination of the vestibulocochlear nerve (CN8) [10, 11], reduced vascular perfusion [12, 13] and increased transmission of pulsatile sound from turbulent blood flow through the cerebrospinal fluid [9]. The IAM internal auditory meatus vascular loop (IVLs) usually arise from the anterior inferior cerebellar artery and may be seen as an anatomical variant in 10% [14]. IVLs and CN8 NVC are optimally evaluated with three-dimensional (3D) fast spin echo or gradient echo high-resolution (HR) T2-weighted (T2w) MRI sequences, and this may be a justification for the inclusion of these sequences in PT MRI protocols.

Most case-controlled studies have shown no difference in the incidence of tinnitus in ears with IVLs [13, 15–18] or CN8 NVC [15, 16, 18, 19]. Whilst some cohorts have included PT ears, they have usually been analysed in combination with non-PT ears [13, 16, 18, 19]. The prevalence of IVLs or CN8 NVC in PT ears alone has only been investigated in small cohorts and without normal ear control groups [9, 10].

Demonstrating an association between IVLs or CN8 NVC and PT would necessitate optimisation of imaging protocols for their detection, analysis and reporting by radiologists, and that clinicians discuss their potential clinical significance and treatment with the patient.

The primary objective was to determine whether there is a difference between the proportion of ears with IVLs or CN8 NVCs on three-dimensional high-resolution (3D HR) T2w MRI when comparing unexplained unilateral PT ears with their contralateral asymptomatic controls. The secondary objectives were to determine whether anatomical co-variates such as IVL angulation and depth influence the likelihood of PT in ears with IVLs, and whether the location of NVC with respect to the transition zone (TZ) or central myelin portion (CMP) of CN8 influences the presence of PT in ears with CN8 NVC.

## Methods

### Patients

Institutional approval (project number 15888) was obtained, and patient consent was waived. This was a retrospective case-controlled cross-sectional study undertaken in a tertiary referral centre. Consecutive patients undergoing MRI for the investigation of PT were identified from a search of a radiology imaging system database between 1/1/2012 to 31/07/2021. The Boolean search terms comprised the keywords tin* AND whoos* OR pulsa*. Patient demographics, laterality of PT, otoscopic findings, other audiovestibular symptoms or diagnoses, and other medical conditions were recorded. The mean air (0.5 kHz, 1 kHz, 2 kHz, 4 kHz, and 8 kHz) and bone conduction thresholds (0.5 kHz, 1 kHz, 2 kHz, and 4 kHz) were recorded from the pure tone audiogram performed at the shortest interval from the MRI study.

**Fig. 1:**
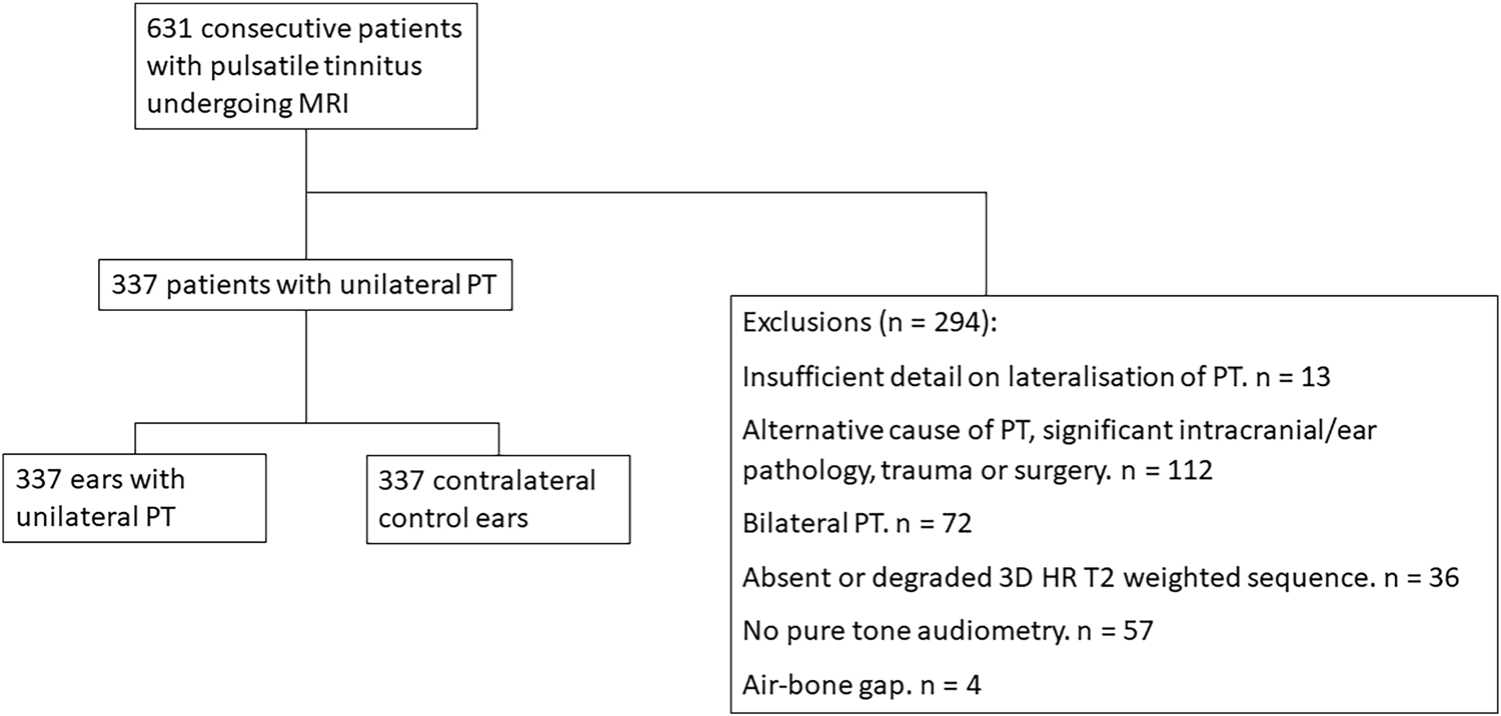
Flow chart of patient selection process. 3D HR, three-dimensional high resolution; PT, pulsatile tinnitus

Exclusion criteria were bilateral tinnitus or insufficient details on lateralisation of PT, absent or degraded 3D HR T2w sequences, alternative causes of PT on imaging or medical assessment, abnormal otoscopy, air-bone gap on audiometry indicating conductive hearing loss [20], absent audiogram, and significant intracranial/ear pathology, trauma, or surgery. The study flowchart shown in Fig. 1 summarises the patient selection process.

### MRI protocol and image analysis

MRI machine details and parameters are shown in Table 1. There were 335/337 (99.4%) studies performed with either 3D HR T2w CISS (constructive interference in steady state) on a 1.5-T Tesla Siemens Magnetom® Aera scanner (276/337; 81.9%) or 3D HR T2w SPACE (sampling perfection with application optimised contrasts using different flip angle evaluation) on a 3-T Tesla Siemens Magnetom® Skyra scanner (59/337; 17.5%).

**Table 1:** MRI parameters

Parameter	T2-CISS	T2-SPACE
Scanner details	1.5 Tesla Siemens Magnetom® Aera	3 Tesla Siemens Magnetom® Skyra
Repetition time	5.36 ms	1000 ms
Echo time	2.41 ms	125 ms
Inversion time	N/A	N/A
Number of excitations	1	2
Refocusing flip angle	62 degrees	100 degrees
Echo train length	1	54
Pixel spacing	0.35 mm	0.5 mm
Slice thickness	0.7 mm	0.5 mm
Matrix size	384 × 512	164 × 320
Field of view	135 × 180 mm	82 × 160 mm
Pixel bandwidth	422 Hz/Px	255 Hz/Px
Number of excitations	1	2

*CISS* constructive interference in steady state, *SPACE* sampling perfection with application-optimised contrasts using different flip angle evaluation

Two subspecialist head and neck radiologists (M.L.C., 5 years’ experience; K.R.S.S., 4 years’ experience) independently analysed the 3D HR T2w axial MRI of the IAMs on a PACS workstation (Sectra workstation, Sectra AB), with the aid of multiplanar reformats. The images were reviewed with standardised magnification and window settings, with scoring and measurements performed bilaterally according to a priori criteria whilst blinded to clinical data.

**Fig. 2:**
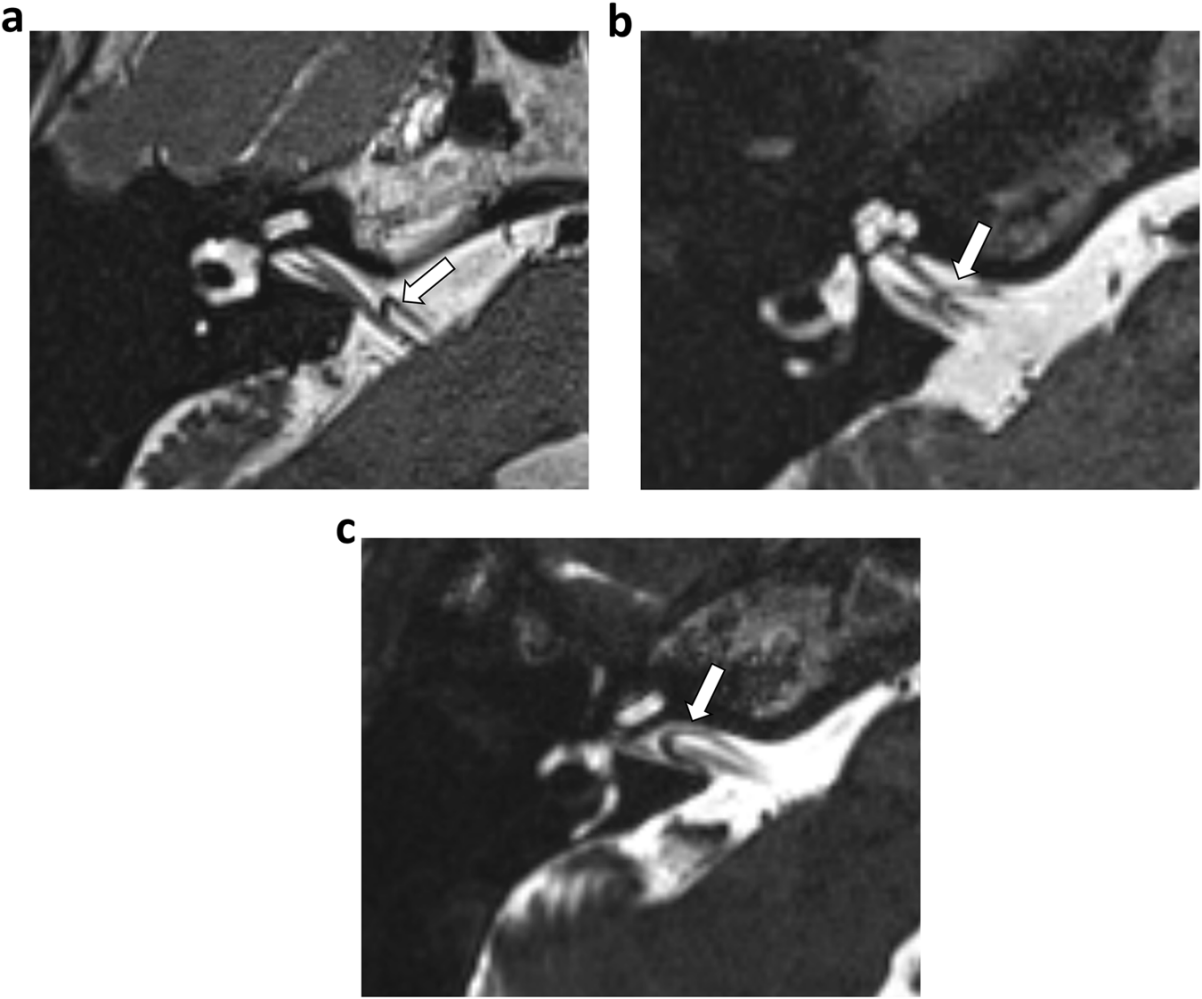
Image analysis for Chavda classification. Unenhanced axial T2w CISS images demonstrate the Chavda classification. VLs are indicated by white arrows. **a** Chavda I, VL located only in the CPA but not entering the IAM. **b** Chavda II, VL entering but not extending greater than 50% of the length of the IAM. **c** Chavda III, VL extending greater than 50% of the length of the IAM. CISS, constructive interference in steady state; IAM, internal auditory meatus; T2w, T2-weighted; VL, vascular loop

**Fig. 3:**
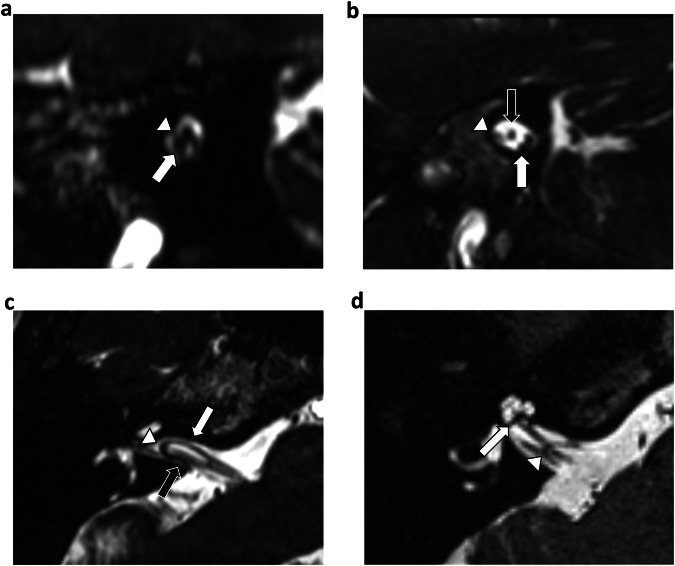
Image analysis for NVC and continuous variables. **a** NVC (sagittal-oblique reformatted unenhanced T2w CISS image). NVC was identified as contact between the VL and the vestibulocochlear or cochlear nerve within the CPA or IAM without a visible intervening normal cerebrospinal fluid signal. The image shows NVC of an IVL (arrowhead) with the cochlear nerve inferiorly (white arrow). **b** An example of the absence of vestibulocochlear NVC within the IAM (sagittal-oblique reformatted unenhanced T2w CISS image). The IAM VL (arrowhead) is located anterior to the facial nerve (black arrow), with normal intervening cerebrospinal fluid signal separating the VL from the cochlear nerve (white arrow) located postero-inferiorly. **c** Loop interlimb distance (axial unenhanced T2w CISS image). Distance (mm) was measured between the proximal (black arrow) and distal (white arrow) limbs of the VL at 2 mm from the VL apex (arrowhead). **d** Loop-fundus distance (axial unenhanced T2w CISS image). Distance (mm) was measured from the VL apex (arrowhead) to the base of the modiolus (arrow). CISS, constructive interference in steady state; CPA, cerebellopontine angle; IAM, internal auditory meatus; NVC, neurovascular contact; T2w, T2-weighted; VL, vascular loop

Imaging criteria and definitions are shown in Table 2. VLs were identified as vascular flow voids communicating with the basilar artery and traversing the ipsilateral cerebellopontine angle (CPA). The location of a CPA/IAM VL was classified according to the Chavda classification, [21]: Chavda I, lying only in the CPA but not entering the IAM; Chavda II, entering but not extending 50% of the length of the IAM; and Chavda III, extending 50% or greater into the IAM (Fig. 2). If more than one CPA/IAM VL communicating with the basilar artery was present, the VL with the higher Chavda classification was utilised for data collection. NVC was identified by the contact of the VL with the vestibulocochlear within the CPA or IAM, or with the cochlear nerve without an intervening normal cerebrospinal fluid signal between structures (Fig. 3).

**Table 2:** Imaging criteria and definitions

Imaging criterion	Definition
Chavda I	VL is located only in the CPA but does not enter the IAM.
Chavda II	VL entering but not extending greater than 50% of the length of the IAM.
Chavda III	VL extending greater than 50% of the length of the IAM.
Loop-fundus distance	Distance (mm) from the VL apex to the base of the modiolus on axial images.
Loop interlimb distance	Distance (mm) between the proximal and distal limbs of the VL at 2 mm from the VL apex.
NVC	Identified as contact between the VL and the vestibulocochlear or cochlear nerve without a visible intervening normal cerebrospinal fluid signal.

*CPA* cerebellopontine angle, *IAM* internal auditory meatus, *NVC* neurovascular contact, *VL* vascular loop

Loop-fundus distance was measured from the IVL apex to the base of the modiolus on axial images. Loop interlimb distance was proposed as a surrogate measure for IVL angulation and was the measured distance between the proximal and distal limbs of the IVL at a consistent 2 mm distance from the loop apex (Fig. 3).

To assess for NVC at the TZ and CMP of CN8 for ears with CN8 NVC within the CPA/IAM, the distance from each NVC to the CN8 origin at the brainstem was measured on coronal oblique images using multiplanar reformats in the line of CN8, with correlation using the axial and sagittal oblique images. The previous anatomical study by Guclu et al identified the most distal part of the TZ to be located in the range of 9.28–13.84 mm (11.50 ± 1.56 mm) from the brainstem with a TZ depth of up to 1.28 mm [22]. CN8-NVC was therefore recorded as within the TZ for distances between 8.0 and 13.8 mm from the brainstem. CN8-NVC at the combined segment of the CMP and TZ (CMP-TZ segment) was recorded for distances of up to 11.5 mm from the brainstem.

For the categorical variables of Chavda classification and CN8 NVC, consensus was obtained with disagreements reviewed by a third radiologist (S.C., 29 years’ experience). For continuous variables of loop-fundus distance and loop interlimb distance, the mean of the two readers’ measurements was utilised for statistical analysis.

### Statistical analysis

Statistical analysis was performed using SPSS® Statistics 27.0 (IBM®).

The inter-rater reliability of the two observers was evaluated with Cohen’s kappa test for the IVL Chavda classification, weighted kappa test for CN8 NVC, and intraclass correlation coefficient (Two-Way Mixed, Absolute) for continuous variables.

McNemar’s paired samples compared the presence of IVLs (Chavda II or III classification) and CN8 NVC in symptomatic unilateral PT ears with their contralateral asymptomatic control ears.

The normally distributed mean air conduction thresholds were compared between the symptomatic unilateral PT ears and the contralateral asymptomatic control ears with the independent samples *t*-test.

For ears with IVLs (Chavda II or III classification), the presence of Chavda II v Chavda III classification, the loop-fundus distance, the loop interlimb distance, and patient age were compared between ears with and without PT. Depending on whether the continuous data was normally distributed according to the Shapiro-Wilk’s test, these comparisons were performed with the independent samples *t*-test (parametric) or the Mann–Whitney *U*-test (non-parametric). The Chi-squared test of independence was used to compare categorical data.

For the ears with CN8 NVC, the presence or absence of contact at the TZ or CMP-TZ region was compared between ears with PT and ears without PT using the Chi-squared test of independence.

A *p* value of ≤ 0.05 was considered to represent statistical significance.

## Results

### Descriptive data of the cohort

631 consecutive patients with PT were referred for investigation with MRI, and the final cohort following application of the exclusion criteria comprised 337 patients with unilateral PT (250 female, 87 male; mean age 47 ± 16 years). Exclusion criteria were patients with bilateral PT (*n* = 72), insufficient detail on lateralisation of PT (*n* = 13), abnormal otoscopy (*n* = 19), alternative medical cause of PT (*n* = 5), alternative cause of PT on imaging (*n* = 42), other significant intracranial/ear pathology, trauma, or surgery (*n* = 46), absent or degraded 3D HR T2w sequences (*n* = 36), air-bone gap on audiometry (*n* = 4), and absent audiometry (*n* = 57) (Fig. 1). The specific exclusions are documented in Supplementary Table 1.

Patient demographics are shown in Table 3. There were 150/674 ears with IVLs and 423/674 ears with CN8 NVC.

**Table 3:** Patient demographics

Characteristic	Value
Number of patients with unilateral PT	*n* = 337
Age (mean ± standard deviation)	47 ± 16 years
Sex	
Male	*n* = 87
Female	*n* = 250
Mean air conduction thresholds in PT ears	*n* = 337; mean 17.2 ± 14.5 dB
Mean air conduction thresholds in asymptomatic ears	*n* = 337; mean 15.7 ± 14.6 dB

### Inter-rater reliability

There was ‘almost perfect’ (> 0.90) [23] level of agreement for IVL Chavda classification (Weighted kappa = 0.91, 95% CI: 0.87–0.94, *p* < 0.001) and a strong (0.80–0.90) level of agreement for CN8 NVC (kappa = 0.88, 95% CI: 0.84–0.91, *p* < 0.001). There was excellent inter-rater reliability for loop-fundus distance (intraclass correlation coefficient = 0.98, 95% CI: 0.96–0.99, *p* < 0.001) and good inter-rater reliability for loop interlimb distance (intraclass correlation coefficient = 0.87, 95% CI: 0.56–0.94, *p* < 0.001) [24].

### MRI findings for VL Chavda classification, CN8 NVC and anatomical variables

**Fig. 4:**
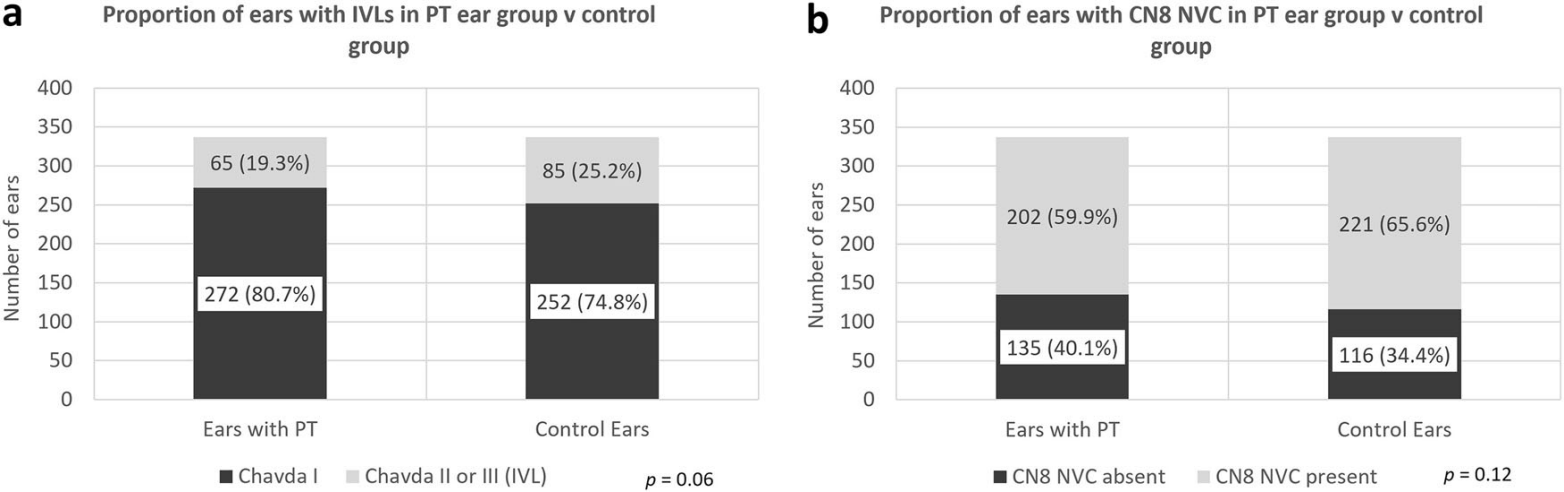
Proportion of ears with IVLs and CN8 NVC in PT ears v controls. **a** IVLs in symptomatic PT ears v contralateral asymptomatic control ears. No significant association was demonstrated between the prevalence of ipsilateral IVLs and PT (*p* = 0.06). **b** CN8 NVC in symptomatic PT ears v contralateral asymptomatic control ears. No significant association was demonstrated between the presence of ipsilateral CN8 NVC and PT (*p* = 0.12). Lower proportions of PT ears were associated with IVLs and CN8 NVCs as compared to the contralateral control ears, however, these did not reach statistical significance. CN8, vestibulocochlear nerve; IAM, internal auditory meatus; IVL, IAM vascular loop; NVC, neurovascular contact; PT, pulsatile tinnitus; VL, vascular loop

**Fig. 5:**
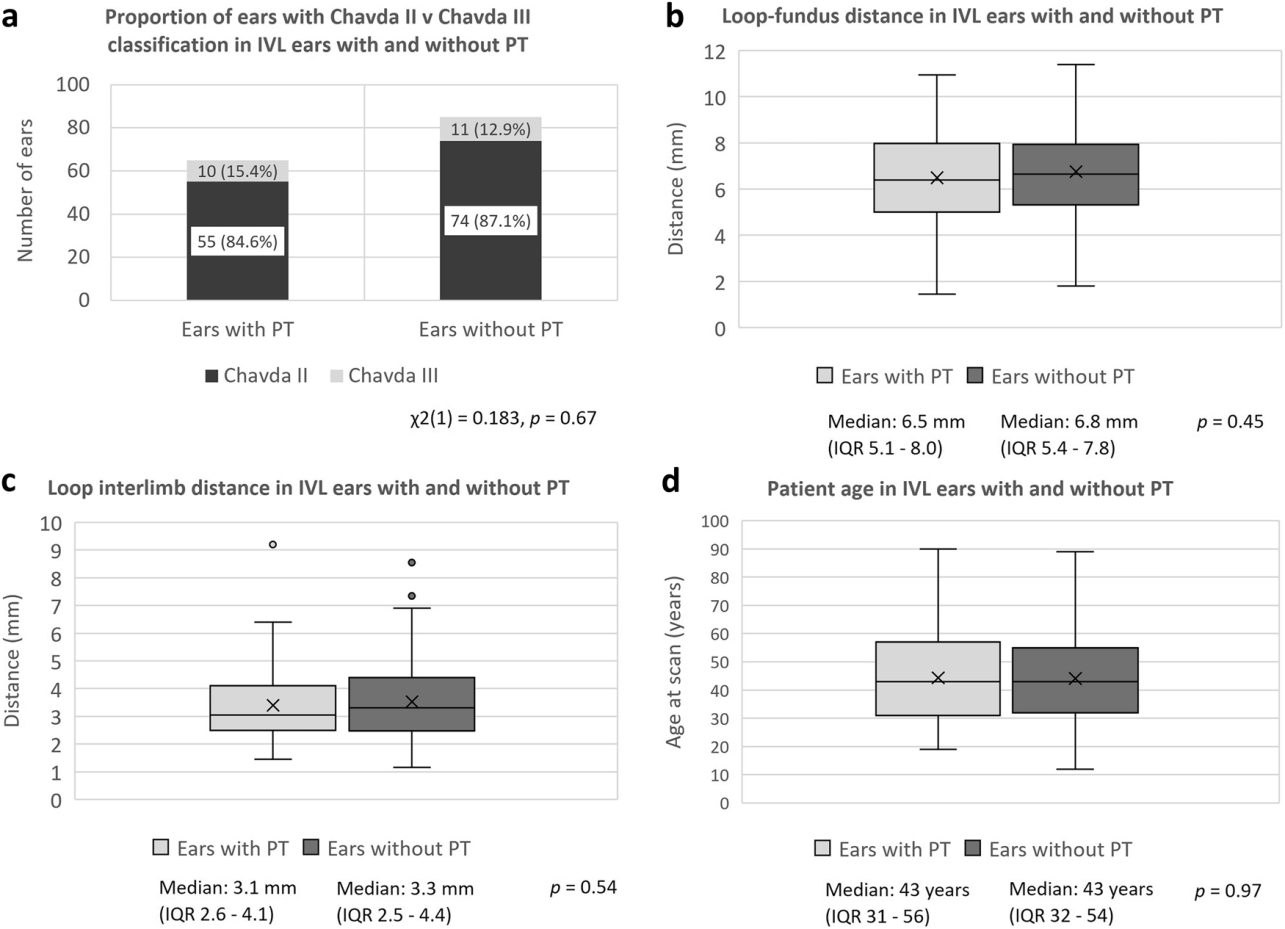
Loop-fundus distance, loop angulation and patient age in IVL ears with and without PT. **a** Proportion of ears with Chavda II v Chavda III in IVL ears with and without PT. There was no difference in the incidence of PT in Chavda II when compared to Chavda III VLs (*p* = 0.67). **b** Loop-fundus distance in IVL ears with and without PT. There was no difference in the loop-fundus distance between ears with PT (median 6.5 mm, IQR 5.1–8.0 mm) and those without PT (median 6.8 mm, IQR 5.4–7.8 mm) (*p* = 0.45). **c** Loop interlimb distance in IVL ears with and without PT. There was no difference in the loop interlimb distance between ears with PT (median 3.1 mm, IQR 2.6–4.1) and those without PT (median 3.3 mm, IQR 2.5–4.4) (*p* = 0.54). **d** Age at scan and PT. There was no difference between the median patient age in ears with PT (median 43 years, IQR 31–56) and those without PT (median 43 years, IQR 32–54) (*p* = 0.97). CN8, vestibulocochlear nerve; IAM, internal auditory meatus; IVL, IAM vascular loop; NVC, neurovascular contact; PT, pulsatile tinnitus; VL, vascular loop

The MRI findings for the VL Chavda classification and CN8 NVC in the unilateral symptomatic PT ears, and ears with and without PT, are summarised in Tables 4 and 5. Bar and box plot charts for all variables are shown in Figs. 4 and 5.

**Table 4:** Chavda classification of VLs for unilateral PT ears and contralateral asymptomatic control ears

Imaging criterion	Unilateral PT ears	Contralateral asymptomatic control ears
Chavda I VL	272/337 (80.7%; 95% CI: 76.1–84.8%)	252/337 (74.8%; 95% CI: 69.8–79.3%)
Chavda II VL	55/337 (16.3%; 95% CI: 12.5–20.7%)	74/337 (22.0%; 95% CI: 17.7–26.8%)
Chavda III VL	10/337 (3.0%; 95% CI: 1.4–5.4%)	11/337 (3.3%; 95% CI: 1.6–5.8%)

*CN8* vestibulocochlear nerve, *IAM,* internal auditory meatus, *IVL* IAM vascular loop, *NVC* neurovascular contact, *PT* pulsatile tinnitus, *VL* vascular loop

**Table 5:** Proportion of IVLs and CN8 NVCs for unilateral PT ears and contralateral asymptomatic control ears

Imaging criterion	Unilateral PT ears	Contralateral asymptomatic control ears	*p* value
IVL present (Chavda II or Chavda III VL)	65/337 (19.3%; 95% CI: 15.2–23.9%)	85/337 (25.2%; 95% CI: 20.7–30.2%)	0.06
CN8 NVC present	202/337 (59.9%; 95% CI: 54.5–65.2%)	221/337 (65.6%; 95% CI: 60.2–70.6%)	0.12

*CN8* vestibulocochlear nerve, *IAM* internal auditory meatus, *IVL* IAM vascular loop, *NVC* neurovascular contact, *PT* pulsatile tinnitus, *VL* vascular loop

### IVLs and CN8 NVC in symptomatic PT ears v contralateral asymptomatic control ears

Of the symptomatic unilateral PT ears, there were 272/337 (80.7%, 95% CI: 76.1–84.8%) Chavda I, 55/337 (16.3%, 95% CI: 12.5–20.7%) Chavda II, and 10/337 (3.0%, 95% CI: 1.4–5.4%) Chavda III VLs. In the contralateral asymptomatic control ears, there were 252/337 (74.8%, 95% CI: 69.8–79.3%) Chavda I, 74/337 (22.0%, 95% CI: 17.7–26.8%) Chavda II, and 11/337 (3.3%, 95% CI: 1.6–5.8%) Chavda III VLs.

There was no statistically significant difference (*p* = 0.06) between the proportion of IVLs in PT ears (65/337; 19.3%; 95% CI: 15.2–23.9%) and that in control ears (85/337; 25.2%; 95% CI: 20.7–30.2%). There was also no statistically significant difference (*p* = 0.12) between the proportion of CN8 NVC within the CPA or IAM in PT ears (202/337; 59.9%; 95% CI: 54.5–65.2%) as compared to control ears (221/337; 65.6%; 95% CI: 60.2–70.6%).

There was no difference in the mean air conduction thresholds between PT ears (mean 17.2 ± 14.5 dB) and the control ears (mean 15.7 ± 14.6 dB) (*p* = 0.17).

### Associations of anatomical variables of IVLs and patient age with PT

In ears with IVLs, the presence of PT was not influenced by the depth of the IVL according to the Chavda classification (Chavda II v Chavda III; χ2(1) = 0.183; *p* = 0.67) or when evaluated by the distance from the IVL apex to the base of the modiolus (median 6.5 mm [IQR 5.1–8.0] with PT; 6.8 mm [IQR 5.4–7.8] without PT; *p* = 0.45). There was no significant difference in the IVL interlimb distance (*p* = 0.54) between ears with PT (3.1 mm [IQR 2.6–4.1]) and without PT (3.3 mm [IQR 2.5–4.4]). There was also no association between patient age and the presence of PT in ears with IVLs (median age 43 years [IQR 31–56] with PT; 43 years [IQR 32–54] without PT (*p* = 0.97).

### Associations of NVC at the TZ or CMP-TZ region and PT in the ears with CN8 NVC

On analysis of the ears with CN8 NVC, there were 143/202 (70.8%; 95% CI: 64.0–77.0%) cases with CN8 NVC at the TZ in ears with PT and 151/221 (68.3%; 95% CI: 61.8–74.4%) cases in ears without PT. There were 166/202 (82.2%; 95% CI: 76.2–87.2%) cases with CN8 NVC at the CMP-TZ segment in ears with PT, and 190/221 (86.0%; 95% CI: 80.7–90.3%) cases in ears without PT. The presence of PT was not influenced by whether or not CN8-NVC was located at the TZ (χ2(1) = 0.303; *p* = 0.58) or whether or not it was located in the central myelinated portion or transition zone (CMP-TZ) segment (χ2(1) = 1.140; *p* = 0.29).

## Discussion

The proportion of ears with IVLs (*p* = 0.06) or vestibulocochlear NVC (*p* = 0.12) did not significantly differ between 337 unexplained PT ears and their contralateral asymptomatic control ears. In ears with IVLs, the presence of PT was not associated with the depth of the VL, as indicated by a Chavda classification III (*p* = 0.67) or the distance of the VL to the fundus of the IAM (*p* = 0.45). Moreover, the angulation of the IVLs, as evaluated by the distance between the two limbs of the VL (*p* = 0.54) was not associated with the presence of PT. In ears with CN8-NVC, the presence of PT was not affected by whether or not the contact was located at the TZ (*p* = 0.58) or within the combined CMP-TZ segment (*p* = 0.29).

Most previous case-controlled studies analysing the presence of IVLs and CN8 NVC have included cohorts of undefined tinnitus that have not distinguished between PT and non-PT ears. These have largely demonstrated no association between either IVLs [13, 16, 18] or CN8 NVC [16, 18, 19] and the presence of tinnitus. A single study showed a significant association between these undefined tinnitus ears and IVLs [13], whilst another reported an association with CN8 NVC [25]. A recent meta-analysis of pooled data from case-controlled studies demonstrated no statistically significant association between the presence of undefined tinnitus and that of IVL (OR 0.90 95% CI: 0.47–1.70) or CN8 NVC (OR 1.15, 95% CI: 0.68–1.95) (*p* > 0.05) [15]. PT ears alone have only been investigated in two previous small studies without asymptomatic control groups. De Ridder et al demonstrated IVLs to be significantly more frequent in 17 unilateral PT ears than in 43 with non-PT [9], whereas Nowé et al found a similar result in their study of 12 PT ears and 46 ears with non-PT [10]. This data was subject to meta-analysis [26] and the authors concluded that contacting VLs were 80 times more frequent in PT compared to non-PT, suggesting a causal relationship and the potential for surgical intervention in some cases. As a result, an IVL continues to be proposed as an aetiology for PT in contemporary imaging guidelines [27, 28]. Our study is the first to investigate the relationship between PT and IVLs or CN8 NVC in a large cohort of PT ears with an asymptomatic control ear group and has shown no association. Whilst it may remain justified to include a 3D HR T2w sequence with PT imaging protocols to identify other aetiologies for PT within the IAM and labyrinth, the present study argues that IVLs and CN8 NVC should not be analysed, reported, or identified as a therapeutic target in the context of PT.

None of the IVL anatomical features investigated were shown to influence the likelihood of PT. De Ridder et al proposed that PT results from pulsatile sound waves being conducted from the VL through the cerebrospinal fluid of the IAM and perineural spaces to the cochlea, which prompted our analysis of the depth of the IVL within the IAM and hence its proximity to the cochlea. Similarly, it has been argued that a sharper turn of the VL may result in greater turbulence and sound transmission [9], and our measurement of the interlimb distance was a surrogate for this angulation. Moreover, it was speculated that VLs may become more tortuous or narrowed with age, however, no association was demonstrated between increasing age and the likelihood of PT symptoms in IVL ears. Other anatomical features of VLs have been explored as potential correlates for PT in previous studies with variable outcomes. Yoo et al demonstrated a relationship between the calibre of an IVL and unspecified tinnitus [13] whilst the CN8 NVC topographic relationship [10, 29] and associated neural distortion [30] have also been proposed as significant anatomical features to analyse.

There are limitations to the present study. Firstly, there was an innate bias in patient selection introduced by the case-controlled study design [31]. Secondly, it would have been optimal to exclude ears with venous variants since they are recognised as a potential aetiology of PT, however, they are inconsistently defined in the literature and some variants are difficult to identify without CT [32]. An alternative approach would have been the exclusion of patients with symptoms and signs of “venous” PT; however, this was precluded by insufficient clinical details. Thirdly, the exclusion of bilateral PT ears may introduce selection bias and limit the applicability of this patient group, however, the inclusion of a matched asymptomatic control ear in unilateral PT may also be considered a strength of the study design. Finally, the study would have benefited from a prior power analysis to ensure that the sample size was sufficient to demonstrate any association between PT and IVL or CN8 NVC and reduce type II error, however, the retrospective study design did not allow us to control the number of ears included. Although criticised as a method for interpreting negative study findings [33], a post-hoc power analysis determined the study to have 78% power for detecting an increase in the incidence of IVL or CN8 NVC by 10% if the incidence in asymptomatic ears was considered to be 30% (α = 0.05). A power value of > 80% is frequently considered sufficient for the determination of the actual effects of research studies.

In conclusion, our data indicate that the presence of an IAM VL or of vestibulocochlear NVC is no more frequent in ears with unexplained unilateral PT than in contralateral asymptomatic ears. In addition, the likelihood of PT was not influenced by the distance of the IAM VL from the IAM fundus or by the angulation of the IAM VL, and PT was no more prevalent if CN8-NVC was located within the TZ or CMP-TZ segments. This would argue against the analysis and reporting of IAM VLs or vestibulocochlear NVC in the context of PT, and it gives the clinician confidence to reassure the patient that these are unlikely to be an aetiology of PT.

## Supplementary information

ELECTRONIC SUPPLEMENTARY MATERIAL

## Abbreviations

3D HR: Three-dimensional high resolution
CISS: Constructive interference in steady state
CMP: Central myelin portion
CN8: Vestibulocochlear nerve
CPA: Cerebellopontine angle
IAM: Internal auditory meatus
IVL: Internal auditory meatus vascular loop
NVC: Neurovascular contact
PT: Pulsatile tinnitus
T2w: T2-weighted
TZ: Transition zone
VL: Vascular loop
