# Supplementary material for: Internal auditory meatus vascular loops and vestibulocochlear neurovascular contact on MRI: Are they associated with pulsatile tinnitus?
Source: Eur Radiol. 2025 May 12;35(11):6745–54. doi: 10.1007/s00330-025-11660-8 (PMC12559041; doi:10.1007/s00330-025-11660-8)
Supplement: Supplementary file 1 — ELECTRONIC SUPPLEMENTARY MATERIAL [file 330_2025_11660_MOESM1_ESM.pdf]

Internal auditory meatus vascular loops and vestibulocochlear neurovascular contact on MRI: Are they associated with pulsatile tinnitus?

ELECTRONIC SUPPLEMENTARY MATERIAL

Eur Radiol (2025) Chong ML, Stephenson KRS, Sultani Tehrani M, Pai I, Connor S.

Supplementary Table 1: Exclusions for abnormal otoscopy, alternative causes of pulsatile tinnitus and other significant intracranial/ear pathology, trauma or surgery.

Exclusion	Number of cases
**Abnormal otoscopy (n=19)**	
Grommet(s)/ventilation tube(s)	7
Chronic otitis media	10
Active otitis externa	2
**Alternative causes of PT on imaging (n=42)**	
Arteriovenous malformation	1
Dural arteriovenous fistula	6
Intracranial internal carotid artery aneurysm	4
Cervical carotid artery aneurysm	2
Severe stenosis of internal carotid artery	2
Confirmed idiopathic intracranial hypertension[a]	16
Otospongiosis	5
Skull base paraganglioma	4
Transverse/sigmoid sinus thrombosis	2
**Other alternative medical cause of PT (n=5)**	
Aortic regurgitation	1
Pansystolic murmur	1
Hyperthyroidism	3
**Other significant intracranial / ear pathology, trauma or surgery (n=46)**	
Ischaemic injury involving auditory pathways	3
Subdural haematoma	1
Encephalomalacia – temporoparietal	1
Cavernoma	1
Facial nerve palsy	2
Chiari I malformation	2
Conductive hearing loss[b]	5
Mixed hearing loss	2
Cochleo-vestibular failure	1
Eustachian tube dysfunction	16
Previously operated semicircular canal dehiscence	2
Intra-axial tumour	3
Traumatic pseudomeningocele	1
Encephalocele	1
Cerebellopontine angle lesion	2
Jugular foramen meningeal cyst	1
Langerhans cell histiocytosis of the temporal bone	1
Meniere’s disease	1
Combined total =	112

[a]: Diagnosis of idiopathic intracranial hypertension as per Dandy Criteria. (1)

[b]: “Conductive hearing loss, defined as 20dB or greater average air-bone gap over three of the following frequencies: 500, 1000, 2000, 3000 or 4000 Hz. Testing of bone conduction thresholds at 3000 and 4000Hz would only be necessary if one of other frequency shows a conductive loss of 20dB or greater air bone gap”. (2)

References:

(1) Wall M. Update on Idiopathic Intracranial Hypertension. Neurol Clin. 2017 Feb;35(1):45–57.

(2) British Academy of Audiology. https://www.baaudiology.org/app/uploads/2023/04/Onward-Referral-Guidance-for-Adult-Audiology-Service-Users-Sept-23.pdf. 2023. Onward Referral Guidance for Adult Audiology Service Users.
